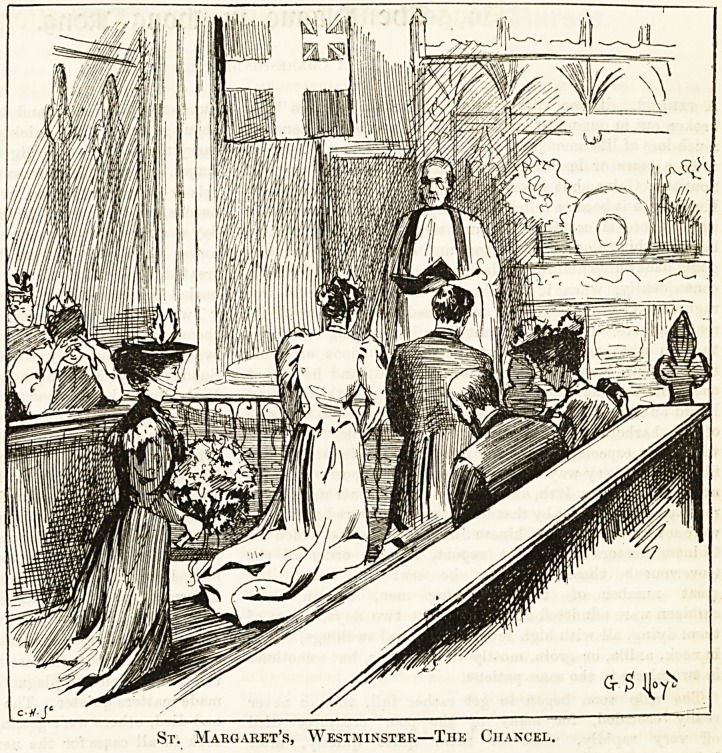# The Hospital Nursing Supplement

**Published:** 1894-07-28

**Authors:** 


### "The Hospital" Nursing Mirror.

Being the Extra Nursing Supplement of "The Hospital" Newspaper.

[Contributions for this Supplement should be addressed to the Editor, The Hospital, 428, Strand, London, W.C., and should have the word "Nursing" plainly written in left-hand top corner of the envelope.]

### News from the Nursing World.

### REGISTRATION OF MIDWIVES.

The reiterated opposition to the proposed registration of midwives has apparently wearied members of the long-suffering medical profession, as well as the general public. The letter of Dr. J. T. Gorham in the *British Medical Journal* deals with the matter very practically, and trained district nurses will be able to endorse his experience that "there are numerous cases in town and country districts where poor women have been maimed for life from the brutal treatment they have met with during their confinements from ignorant women who are allowed to practice as 'midwives' by the present law. . . That this great hardship which falls upon the poor, and only the poor, can to a great extent be remedied by the proposed measure to register midwives," and "that our profession, from the action of a minority of over-zealous members, may be charged with greedy and selfish motives in resisting a measure whose object is to confer a great boon on poor people."

### EMPLOYMENT OF EPILEPTICS.

In connection with the National Society for the Employment of Epileptics, a temporary building is shortly to be opened, which will contain accommodation for eighteen or twenty male patients. The permanent colony of cottage homes at Chalfont St. Peter's is not yet begun, but will probably be commenced in the course of a few weeks. Out of a large number of applicants selection has already been made of cases for admission. It is stated that funds are still urgently needed for the work, and especially for the erection of buildings for female patients. The committee decide the admissions, the voting system not being adopted by the society.

### A PLEASANT DAY.

The Morley House Seaside Convalescent Home has increased in size and usefulness until it now contains eighty beds. The house is pleasantly situated at St. Margaret's Bay, and the annual excursion on the 14th inst., consisting of members of trade, benefit and friendly societies, resulted in a pleasant visit to the institution. A good many beds are endowed by different London firms which in this way secure them for convalescent patients of various trades or callings. "Caxton" is the appropriate name of the ward allotted to printers, another is devoted to the use of postmen, whilst the City Police and members of the Hearts of Oak Society and many others are equally wisely provided for.

### NURSE DOLLS AT STAMFORD.

The nurse dolls have been much appreciated at Stamford, where they assisted at a jumble sale arranged by Miss Sewell and Mr. J. S. Loweth, in aid of the funds of the Stamford branch of the Queen's Jubilee Institute. Mrs. Blackmore, Queen's nurse, presided over The Hospital Collection of nurse dolls, which commanded considerable attention. Nearly £24 was raised by the jumble sale, all the stalls being cleared within two hours and a half of the opening, and the undertaking was extremely well managed and satisfactory throughout.

### NIGHT NURSING.

The appointment of a second night nurse at Chester Workhouse Infirmary, referred to in another column, appears to be already arranged for. Mr. Butler and Mr. Rowe Morris failed to see "any justification for an increase in the nursing staff," according to the report in the local press. But Mrs. Douglas promptly inquired whether it was right to leave these helpless old people at the mercy of a casual pauper, and drew attention to the absolute need of a night nurse in each block, the two buildings being situated at some distance apart. She also remarked with some spirit that it was a question to decide by "using our own common sense." The master showed that there were between seventy and eighty old and infirm people needing constant attention, and the recommendation was eventually carried. The Chester Guardians are to be congratulated on their decision, and also on having associated with them a lady who not only possesses common sense herself, but advocates its use by other people.

### MASSAGE.

Recently certain existing evils have been brought to notice as to the large and dangerous abuses now existing in establishments ostensibly advertised as massage-rooms. Probably this movement will result in the suppression of the objectionable advertisements, and also of the establishments referred to. In the meantime, it behoves everyone who aspires to become a masseuse to make exceedingly careful inquiries before beginning a course of lessons, and still more thorough investigations before entering upon an engagement to work in any establishment whatever. No one will rejoice more than the honest and conscientious masseur or masseuse at the exposure and stamping out of those practices which are said to be cloaked under the familiar word "massage." A suggestion that the art of massage, as countenanced and prescribed by physicians, should be used on behalf of male patients by rubbers of the same sex only, is a commendable one; but at present it seems doubtful whether there is an adequate supply of qualified masseurs to meet the demand.

### QUEEN'S NURSES AT DROGHEDA.

For the last eight months the sick poor at Drogheda have had a trained Queen's nurse provided for them by Mr. Dickson's thoughtful liberality. The experiment has turned out a very successful one, the patients and their friends proving most appreciative of the services rendered; and it is, therefore, desirable that this temporary arrangement made by Mr. Dickson should be continued permanently by the citizens. To this end, a well-attended meeting was held in the Mayoralty

House last week, and the Mayor, who presided, spoke warmly in favour of the scheme. Mr. Dickson explained the nature of the work which had been so successfully carried out, and laid stress on the fact that not only actual nursing of a patient, but also valuable instruction to the friends, was given by the trained district nurse, whose object it was to teach the poor how to attend intelligently upon their sick relatives between the nurse's visits. Miss Dunne, inspector of the Irish branch of the Jubilee Institute, gave an excellent address. She was received with much applause, and her admirable remarks on district nursing gained the attention they merited. There seems every prospect of other Queen's nurses eventually joining the one who has already done such good service, for it seems to be unanimously agreed that, in so populous a district as Drogheda, full employment could be found for three nurses if funds were forthcoming to support them. However, a good beginning has been already made.

### LEPERS IN NORWAY.

The natural surroundings of the Leper Hospital at Molde are exceptionally fine. Grand views of the Romsdalhorn, of wonderful blue water, and a most perfect garden, are all within reach of the inmates; but, says a contributor to the *World* who has described a visit paid to the place, "no patients ever leave the hospital when once they have been placed inside," so even the beauties of nature must sometimes prove poor consolation to these permanently incarcerated sufferers. All modern appliances are provided in the house, and the kitchen is quite a model one. The cooking, done by steam, is all according to the most approved modern methods. All patients capable of work have employment provided for them—a truly merciful provision for these terribly afflicted persons, for whom their spinning, mat-making, and gardening perchance serve to render the hours a trifle less monotonous.

### MALE NURSES.

What appears from the report to be a very just encouragement to the male nurses at the Tenon Hospital was recently made through the instrumentality of the chief surgeon, Dr. Félizet. Sixteen of the men received certificates on the conclusion of the courses of medical and nursing lectures given to them, and a more substantial reward was not lacking, a sum of money being placed in the savings bank for their personal benefit as a practical reward, which certainly could not fail to be appreciated.

### NURSES IN FRANCE.

During the typhoid fever epidemics of 1893-94 in the barracks at Lure, Madame Berges, better known as Sister Marie Frederique, was conspicuous for her devoted attention to the sick, and for the remarkable courage with which she faced and overcame difficulties. A medal has recently been awarded to her by the Minister of War, and the remembrance of her good work will long live in the minds of those to whose welfare she devoted herself. For thirty-three years Madame Grénier has faithfully served the sick soldiers in the Auch Military Hospital. Her title of Sister Emilie is familiar to all residents, and many rejoice at the presentation made to her lately of a gold medal in acknowledgment of her zeal and efficiency.

### MADAGASCAR.

The Friends' Foreign Mission Association has just published a report of the work of their Medical Mission at Antananarivo. There are four dispensaries for out-patients, situated at Analakely, Isoavinandriana, Ambohipotsy, and Flazaina. At Isoavinandriana in-patients are received. Miss C. L. Byam, M.R.R.C., is the Lady Superintendent and teacher of nurses. She gives instruction in bandaging, details of nursing, physiology, nursing of special cases, midwifery, and Bible classes. The report of Dr. Fenn gives abundant evidence of the difficulties which the mission has had to encounter during the past year. The great storm in January, 1893, did such destruction to the hospital premises, particularly to the quarters of the nursing staff, that it was six months before the damage was repaired, the wet season causing delay in the work. During this time Miss Byam and her staff had to use a ward, cleared for the purpose. Although this was the best arrangement under the circumstances, all were glad when the time came for returning to their proper rooms. The number of these had been added to and other improvements and additions were introduced in the course of the rebuilding. The Lady Superintendent's report shows that nursing is quite a fashionable profession in Madagascar, and the applications of candidates far exceed the vacancies for probationers in the hospital. The examination of the probationers at the conclusion of their course of instruction was conducted by Dr. Ralarosy, the native house surgeon, and the results were eminently satisfactory as regards the majority of probationers, "but for a few days afterwards it was somewhat trying, as those who had not passed were in tears, and those who had passed were miserable because their friends had failed." A hospital has now been erected at Vonizongo, and a nurse from Isoavinandriana is to be in charge of it. Altogether nursing in the Island of Madagascar appears to be making rapid progress, and to be thoroughly appreciated and helped forward by the natives.

### LITTLE NOURISHMENTS.

"What have you fed him on lately?" asked the doctor in the out-patient department. "He's had his little nourishments reg'lar," is the familiar response in a slightly defiant tone. "He allus has just the same as me and his father." Well may the doctor sigh, realising that the half-eaten, rotten plum in the baby's tiny hand may be taken as a sample of his "little nourishments." But even admitting the child as an in-patient will not altogether protect him unless the nurse's vigilance is incessantly exercised. Many a little delicacy is surreptitiously administered on visiting days—even tinned foods being now procurable on trucks invitingly near to the entrance, all ready opened by the importunate vendors. The patients' friends apparently consider the rules against bringing in food are more honoured in the breach than the observance, and are inclined to act accordingly.

### SHORT ITEMS.

There was only one applicant for the post of head nurse at Downpatrick Workhouse, and the *Irish News* reports that she "was elected unanimously." As she has been assistant nurse in the institution the Guardians have had a fair opportunity of judging of her abilities, and her election to her present post was probably a foregone conclusion.—The institution of a district trained nurse for Dursley is now being energetically taken up by a local committee of ladies.—The late matron of the Newton Abbot Workhouse, Ann Mance, died of heart disease somewhat suddenly last week.

### On General Nursing.

By ROWLAND HUMPHREYS, M.R.C.S., L.R.C.P.Lond.

### XXII.—TYPHOID FEVER.

TYPHOID fever is one of those complaints in which nursing skill displays itself to the best advantage, because, till quite recently, no general attempt was made to cut it short, it was simply allowed to run its course. Nowadays we recognise that the disease is one in which a specific germ plays a very considerable, if, indeed, not the leading part, and therefore the knowledge we possess of antiseptics is being applied not only to the prevention of the disease, but also to the treatment of those persons whose bodies have been made the receptacles of, and germinating places for, the bacilli of typhoid. The typhoid bacillus having obtained entrance to the intestinal tract of such a person (the bacillus being derived from some excretion of another person suffering from the same complaint), it obtains a lodgment there, and begins to develop. The period of time required for its proper development is from one to two weeks, usually the latter period. Its growth is marked by a feeling of not being well, with perhaps a little diarrhœa, headache, or sore throat, but there is nothing at all characteristic about these symptoms. The first definite, or more usually the first indefinite, but marked symptom occurs at the commencement of the illness, and is often a headache, and a sense of languidness always accompanies it, with more or less fever. The very indefiniteness of the condition is one of its characteristics, and when an ill-defined complaint runs on for a week or more, the chances are greatly in favour of its being the complaint under discussion. The patient presents a typically languid, depressed appearance, and the appetite falls off, the tongue becoming coated.

At the end of the first week the temperature, which has been rising, in a stair-like manner, two degrees at night falling one in the morning, reaches 104 or 105 Fahr., and the patient becomes confined to bed, sometimes from sheer inability to get about, at other times from some complication as pneumonia. Diarrhœa now begins to be marked, the stools varying in number from one or two a day to every hour. The spleen, an organ situated under the ribs high up in the abdomen on the left side, becomes swollen, so as to be considerably enlarged. This enlargement is always present in fevers, but appears to be marked earlier in typhoid than in other fevers; a slight yellowish tint appears on the skin, and seems to be most marked on the hands and feet. The author has observed that the "tache cerebrale" is present on the abdomen at an early stage of the complaint, and as time elapses so the "tache" can be made out higher and higher on the body, at length reaching the forehead. The "tache" can easily be demonstrated by slightly scratching the skin, either transversely or horizontally, when a red mark appears which soon fades, only to reappear and again fade, and again reappear, becoming fainter each time, until after two or three minutes it disappears altogether. Tremulousness if the fingers be separated and the hand held out becomes marked. The tongue on being put out likewise exhibits this trembling, and as the complaint progresses so the tremulousness increases, and in bad cases, where the debility is extremely marked, the condition becomes very pronounced.

The pulse very early, as a rule, becomes "dicrotic" or "hyper-dicrotic," that is, it feels as if each beat were divided into two parts, the vessel being very soft and compressible, and very relaxed. The respiration is increased in frequency, and there is often a cough. The cough is a very early sign, the lungs being often inflamed, and at the first the case is often mistaken for one of acute pneumonia, but there is a peculiar appearance about the patient which, taken with the presence of light-coloured di[illegible]rrhœa, and the appearance of the temperature chart, is indicative of the typhoid origin of the lung trouble.

The diarrhœa is caused by the increased peristaltic action of the larger, and probably of the smaller intestine, so that food taken by the mouth is hurried through the intestinal tract, and reaches the rectum almost before it has had time to be altered by digestion. The food, indeed, often comes through unaltered, so that curds, pills, and such like make their appearance in the fæces. The cause of the diarrhœa is, at any rate in part, due to the irritable condition of the mucuous lining of the intestine, especially of the small intestine. Certain structures called Peyer's Patches, and the solitary glands which are found in the small intestine throughout its whole length, become inflamed through absorbtion (their special office) of the poisonous substances made by the bacilli of typhoid. These substances irritate the structures which absorb them, and the latter becoming inflamed, ulcerate, and the ulceration may extend to, or through the peritoneal coat of the intestine, in the one case setting up peritonitis, in the other perforating the intestinal wall, and allowing the contents of the bowel to escape into the peritoneal cavity, acute general peritonitis resulting. It is apparent that there is an urgent neccessity for seeing that all food given by the mouth is so finely divided that it will be in its greater part digested, that is, rendered liquid and absorbable by the intestinal wall before it has proceeded far down the bowel. Not unfrequently a milk diet pure and simple is ordered, but the milk must never be administered undiluted, because one of the properties of the gastric juice is to curdle the milk, curds and whey being formed, and these curds, if the milk be not thickened nor diluted may, in the state of irritation of the bowel, be hurried through it at such a rate as to appear unaltered in the fæcal discharges. This means, of course, that they have passed over the irritated, inflamed, and possibly ulcerated or gangrenous surfaces of the glandular structures above mentioned, at the risk of detaching the sloughs and setting up uncontrollable hæmorrhage if a blood vessel has been injured in the ulcerative process.

The same applies to food which contains anything indigestible. It is necessary to be extremely careful to administer only food which will not tend to form hard lumps, and in which no material will remain to pass undigested through the bowel. This dieting is not easy to carry out, for the mucous membrane is not in a state to absorb much food, and the processes of digestion are not properly performed.

The digestion of animal food is carried out either in the stomach by the hydrochloric acid formed there or by the pepsin, the digestive ferment of the stomach, which has the power of transforming the lumps of meat which enter the stomach, and which cannot as such be taken up by or made use of in the body, into liquid and easily absorbed nourishment. The digestion of the meat is also carried out in part by the pancreatic juice, which likewise has the power, amongst others, of dissolving up the meat which remains unaltered after passing through the stomach. Under the head of animal diet are included, of course, eggs, milk, fish, meat, and such like. The more finely divided the food is the more readily is it digested, and the less likely are masses of food to remain undigested and so pass through the bowel.

The pancreatic juice has likewise the power of digesting the starchy food which has been taken. It dissolves up the starchy particles, turning them into a liquid form of sugar, which can be easily taken up by the lymphatic vessels contained in the coats of the intestine and thus conveyed to the general circulation.

### Suggestions for Intending Probationers.

By Nurse Mildred.

I intend the following hints to be chiefly for those whose ambition it is to wear the nursing garb, and to spend their future (or at least some part of it) in caring for the sick and helpless.

And a good thing it is, no doubt, for the poor sick folk that there are people anxious and willing to be trained to aid them. But my advice to these would-be nurses is, Wait a bit, don't be in too great a hurry; think well over it, both for your own sake and for the sake of those who will be so dependent on you. Don't imagine that life will be "a bed of roses," though healthy, honest work does give pleasure, and all able-bodied women, with no special home ties, ought to be independent. Yet decidedly in hospital life there are many difficulties to be overcome, disagreeables to be faced, anxieties and responsibilities of a kind not met with in the same degree elsewhere

Then there is the personal point (which, of course, might apply to many branches of work) of complete severance from home life and home interests. It is difficult, almost impossible, if hospital work has to be given up, to pick up the threads and live the home life once more. The work is so absorbing, perhaps so narrowing, there seems little or no time for intercourse with the home people, and this makes a breach difficult to bridge over. Don't go to hospital just because you must earn your daily bread, and perhaps you will have a better position than in any branch of domestic service. Don't take up nursing if you are feeling tired of home, because it is quiet or you don't get on there, or want a fresh excitement, or to have more freedom of action than most girls have at home. Don't think of being a nurse unless you are confident of a certain amount of natural fitness.

But you may ask, How shall we know if we possess the fundamental elements on which the structure of the good work is to be built?

Let me tell you. If you are a selfish, self-indulgent, idle, helpless woman, with a temper little under control, you are unfit for hospital life. On the other hand, if without being perfect (and who is so?) you have a true desire to help others, realising the necessity of being truthful, conscientious, and patient under provocation, and, last, but not least, if you are strong and healthy, and able to use your hands, you are quite justified in asking for the theoretical knowledge and mechanical skill that hospital training offers. No power, either intellectual or mechanical, will come amiss in your nursing life.

I do not say that individuals who have the earnest wish to become helpful, and to gain self-control, cannot do it in hospital; but time is lost there in acquiring what might have been learnt before, and experience thus gained is apt to be bought at the patients' expense. Well, supposing that, after careful consideration, a girl applies for a vacancy in some hospital. If she has no nurse friend to advise her, then difficulties arise. If only twenty years of age, she is too young for admission into most adult hospitals. It will be easier to get into a children's hospital, where there is certainly much to be learnt. But at twenty-five or older it would be a waste of time to have special children's training, as it is now considered necessary to hold a certificate of three years' training from a general hospital.

It is by no means easy to obtain a vacancy as a probationer, strange as it may seem. A nurse's life is certainly often hard and the pay small considering the immense responsibility falling upon those holding responsible posts in their profession. Still, I hope that anyone facing the desired future in the right spirit may be one of the successful candidates.

A personal interview is generally considered desirable. It seems a terrible ordeal to look forward to (well do I remember my spirits quaking and my heart sinking when I went through the experience). But it is worse in anticipation than reality, as the matron is sure to be pleasant even although she may not have a vacancy nor think the applicant suitable. If unsuccessful at the first, then the aspirant can try elsewhere. There are plenty of other hospitals, and it is sometimes easier to get a vacancy as a paying probationer, as there are fewer applicants for these.

Paying probationers have in most hospitals only to sign for one year, but it is no advantage to those who wish to make nursing their profession, and to my mind it is a decided waste of money. The fee would probably be about £1 1s. per week, the probationer having to provide her own uniform and pay her laundry expenses. Probably some time may elapse between getting the promise of a vacancy and being required at the hospital.

The best use should be made of this time, being as much in the open air as possible, and by acquiring all practical knowledge possible—ordinary everyday practical knowledge. All special theoretical work and studies can be taken afterwards, and will be taught systematically in hospital, the theory going along with the practice. This makes the most satisfactory result, according to my experience.

Then about clothes. As few should be taken as possible. If kept on, uniform is given for ward use. A perfectly plain black dress can be worn until uniform is granted, or what is much better, three print or Galatea dresses made quite plainly, like a servant's morning print, plenty of cuffs and collars (the latter of the Eton or St. James's shape), and shoes of comfortable sizes with square toes, and with heels, but these must be low ones.

Caps and aprons will be given from the beginning to an "ordinary probationer," and to a paying probationer patterns of both will be supplied, which, of course, must be copied exactly; four caps will be sufficient and about a dozen aprons. For the rest, one dress, &c., for visiting friends is needful, and also plenty of underlinen which does not take much room nor get out of fashion. A few books, pictures, and ornaments will make the small room more home like, but it is not well to take too many, as they will have to be dusted. Many hospitals now have separate bed-rooms for their probationers. This is a great comfort, for privacy in hospital life is valuable. It is a pity that want of funds to build sufficient accommodation for the nursing staff makes this luxury impossible in some hospitals.

Then the would-be nurse arrives at the door of the hospital, and I will leave her there, to enter on the line of duty she has chosen. Let it be said of her when it ends, "she hath done what she could."

### Chester Workhouse Infirmary.

That the infirmary be made into a separate department under a trained superintendent, is the advice of the *British Medical Journal's* Special Commissioner in concluding a report on the Workhouse at Chester. The recommendation is certainly a sound one. At present one night nurse is responsible for about 160 patients distributed in two separate blocks of buildings. By day there is only one nurse in each block, and pauper assistants. The wards are small and home-like, bright and comfortable. But the supervision of 80 persons thus located is a heavy task for a single nurse. The little children have, through the private gift of a guardian, good cots and other comforts, but they have only a pauper attendant. The isolation wards have no proper nurse. The report shows the airing grounds to be as unattractively bare as at other similar institutions, but the bridges which connect the blocks are utilised for the patients after a very pleasant fashion. Attention is also drawn to the need for bath-rooms, as the present primitive arrangement consists in moveable ones placed on landings, behind screens, is hardly suitable for the sick and infirm.

### Miss Hampton in England.

It was singularly appropriate that the marriage of Miss Isabel Hampton with Dr. Robb should take place at St. Margaret's, Westminster, which has long been a favourite resort of their country men and women. The fine west window was placed in that beautiful old church by a number of Americans, in memory of Sir Walter Raleigh, whose decapitated body was buried in the chancel after his execution in Palace Yard. The late American Minister, Mr. J. R. Lowell, gave the inscription for the window, which reads as follows :—

The New World's sons—from
England's breast we drew
Such milk, as bids remember
whence we came ;
Proud of her Past, from
which our Present grew,
This window we inscribe
with Raleigh's name.

St. Margaret's has been the scene of many strange incidents, and it was in this, "the Church of the House of Commons," as it was called, that Hugh Peter, in addressing a congregation, urged that Charles I. should be brought to punishment. On the same spot Case, later on, abused Oliver Cromwell to his face, asserting that he was willing to sell the three kingdoms "for filthy lucre."

St. Margaret's, Westminster—The Chancel.

Cliveden.

Two mansions bearing this name have been successively destroyed by fire, and the present building was erected in 1851. It was designed by Sir Charles Barry, and is a well-known landmark to all lovers of old Father Thames. The Duke of Westminster always allowed this magnificent palace to be inspected by visitors, except when the family chanced to be there Many visitors can look back gratefully to sunny afternoons when certain of the Duke's people were permitted to supply them with hot water to facilitate tea-making in their boats. The present owner, Mr. Astor, kindly placed a suite of apartments at Cliveden at the disposal of Dr. and Mrs. Robb during their honeymoon, and we give a view of the exterior of this ideal English mansion. The terrace is 430 feet in length, and commands one of the most extensive views on the river. The woods at Cliveden are particularly fine, and overhang the water for a couple of miles.

Cliveden.

### The Plague in Hong Kong.

(By a Correspondent on the Spot.)

A terrible disease known as the "Bubonic Plageu" has broken out in our midst, and has caused, and is still causing, much loss of life amongst the Chinese population. It appears to be a more or less frequent epidemic in several parts of Southern China, but has never broken out before in Hong Kong since it became a British colony, over fifty-three years ago. There is constant and uninterrupted communication between this colony and Canton, and especially a continual going backwards and forwards of the coolie class of Chinese, consequently, when it was known that this sickness was raging at Canton there was an immediate prospect of its reaching here. One doctor went from Hong Kong to Canton to learn what he could about the disease. Soon after his return some suspected cases were reported, and he was at once able to diagnose them. At a meeting of the Sanitary Board on May 10th, it was decided to bring over to this side of the harbour the Government hospital hulk "Hygeia," which was especially built for isolating cases in, and is well fitted up in every way. It was towed over opposite to a disused wharf on May 12th, and by two p.m. patients were being received on board, for by that time it was discovered that there were several cases in the Chinese Junglitah Hospital which the Chinese doctors would not report, but by order of the Government these had to be sent on board. A great number of these suffering men, women, and children were admitted during the next two days, many of them dying, all with high fever and typical swellings, either in neck, axilla, or groin, mostly in the latter, but sometimes in two places in the same patient.

The ship soon began to get rather full, though never really crowded, for many of the poor creatures died off very rapidly, some of them quite quietly, after being comatose for a time; others were wildly delirious, and a few had severe convulsions. Most of the swellings were painful, painting over with glycerine and belladonna frequently seemed to give ease. In a few cases the pain was so great that an injection of morphia was ordered. It soon became necessary to provide more room for the rapidly-increasing number of patients, and on May 14th the new police station was cleared out at the extreme end of the town, and got ready for 100 patients. Patients were received there from the "Hygeia" in about three hours, and all the women were removed out, as the English sisters undertook the work at this station. The "Hygeia" was managed by wardmasters and a good staff of Chinese men, it being in excellent working order. The new quarters at Kennedy Town required organising, for nothing would tempt any Chinese women to remain and help the sisters to nurse, though a very liberal offer of dollars was made (very dear to the heart of the Chinese). For the next few days the work was tremendous in every branch; volunteers were asked for to assist the police, and sanitary inspectors to hunt up the sick in their homes. There was a grievous propensity amongst their friends to hide them anywhere—under low beds, in cupboards, on roofs; some sufferers being even found down a trap-door in the floor of a theatre. The Chinese doctors, merchants, and those of a better class put every possible obstacle in the way of the Europeans who were doing all they possibly could to help these poor suffering creatures. They posted absurd placards, telling these ignorant, superstitious people that the plague was due to the foreigners having made the tramway up to the Peak some years before, and to their having made a new reservoir; by all sorts of abominable reports they endeavoured to incite riots.

The daily returns showed alarming increases in the number of admissions and deaths, and great difficulties arose about getting coffins quickly enough, or sufficient coolies to carry the bodies or to dig the graves, even at exhorbitant wages. Then there were so many Chinese customs to prevent this or that being done, which made the work very much harder, and much ill-feeling among the Chinese. One day a strike was threatened amongst the shipping coolies, unless their people were left alone in their houses to be treated as they chose or left to die, and to be buried or not as suited them best. However, the gunboat "Tweed" was brought close to China Town, which frightened them, showing that the English would not give in to them in everything, and they went to work again next day. On Sunday, May 20th, there was a large meeting at the Tung Wah Hospital of the committee and about 300 other Chinese to discuss the state of affairs, to ask that the house-to-house visitation should be stopped, and that their people should be attended by native doctors in a building near Kennedy Town Hospital, a disused glass works. Their chairman, who though a Chinaman was a member of the Sanitary Board, was mobbed as he was leaving the hospital by hundreds of women, and was obliged to go back until a guard of mounted Indian police came to escort him to his hory (business house) which had been attacked. The town was in a great state of ferment, and it was scarcely safe for Europeans to go in some parts. The police were stopped in their house-to-house visitation, and soldiers had to be sent to help them. The request for Chinese to start the glass works as a branch to the Tung Wah for plague cases was at once granted, and made matters quieter. The worst cases at Kennedy Town had died, others were getting on, and so as the glass works took in all cases for the next week, Kennedy Town quieted down and the patients seemed happy and comfortable. Words cannot describe the dirt, wretchedness, and misery of the Glass Works' Hospital, managed by Chinese, although English doctors went in and out when they chose, and tried to keep a sort of discipline (but, of course, not treating or having anything to do with the sick, according to orders). The patients were mostly on the floors, a few on tressel beds, all in their own dirty rags, some with friends looking after them, others without, no one to attend to them properly; the poor creatures dying without anyone taking notice of them. Two police sergeants were always at the entrance taking the names of patients admitted and of bodies taken out, and much difficulty was experienced in getting the latter buried promptly. On May 21st, the General was asked for help, and he immediately offered 150 men a day; these were divided into two batches, under officers, each lot working three hours a day, either morning or evening, cleaning and whitewashing the houses whence plague-stricken patients had been removed. They all volunteered their help, and truly noble it was of them. More revolting and trying work it would be impossible to conceive.

English people can hardly imagine how large a number of people live in one small house (sometimes as many as sixty), amidst dirt and rubbish which has accumulated for years, and has to be literally dug out before any cleaning can be started. On May 29th it was decided that all the patients from the "Hygeia" should be sent to Kennedy Town, where there was then room for them, as only an occasional Portuguese or Indian was being taken in, and but a few Chinese remained under treatment. It had been thought that Europeans ran no risk of taking the disease, but the same day a soldier was stricken and was sent on board, two more the next day, then two more and an officer, who had been working very hard, and one of the first who had volunteered. This caused much alarm, as it was felt that none of those working in the midst of the disease were safe, and the confidence of European immunity was shattered. Our hands were indeed again full, and great anxiety was felt by everyone. In spite of every effort the brave officer and a sailor died on June 4th. The other soldiers (ten in all) are getting on very well, and no more Europeans have been attacked. Some Japanese have been admitted to Kennedy Town, and now a better class of Chinese and Indians appear to be attacked. There are generally about thirty-six to forty patients at Kennedy Town, many have been discharged other are convalescent, but a number have died. At the glass works as many as 200 were under treatment at a time.

The Chinese authorities at Canton sent down large junks, very well fitted up and quite clean, to take back as many sick as could be sent—about 130 went, only eight dying on the voyage. This emptied the glass works, and the Chinese have now a much better building provided for them by the Government, and supervised as to cleanliness by two naval and two military orderlies lent for the purpose. After six weeks it appears that the plague is decreasing, but when we remember the very large number of deaths (over 2,000) and the people who have fled from the colony (probably 100,000), it is doubtful if the percentage is really lower, though the number remaining under treatment is very much less, and also the daily number of deaths. At the commencement medical help was given by the Navy and Army, a surgeon from each having been daily very hard at work the whole time. Without them it would have been impossible for the Government medical officers to do nearly all that has been accomplished; and some blue-jackets have rendered most valuable help with the launches and in other ways.

### Hints on Disinfection.

(By a Nurse.)

The windows and doors being shut, the room must be kept full of carbolic acid fumes for twelve hours by means of one of Savory and Moore's Vaporisers. Then the windows must be opened for twelve hours before cleaners and workpeople are admitted. The floors, paint, and furniture must be well washed with a carbolic solution, 1 in 80, or with Calvert's carbolic acid soap. The ceiling and walls should then be limewashed, after which the cleaning process should be repeated. While the work is in progress a good fire should be burning in the grate and the windows kept open. The full directions are given with the apparatus from Savory and Moore, London, and it is the best for disinfecting a room that I know of. I have used the vaporiser when preparing for an ovarian operation.

*** We should be glad to hear from other nurses of the methods they follow when the responsibility of disinfecting a room is left to them.—Ed. *T. H.*

### Entertainments.

A Famous Picture.—A magnificent picture of the marriage of T.R.H. the Duke and Duchess of York is now on view at Mr. Mendoza's Gallery, King Street, St. James's. The picture was executed by command of the Queen, and is well worth going to see, being quite as excellent in portraiture and execution as a former picture by the same artist of the Queen and all the Royal Family at Windsor on the occasion of her Majesty's Jubilee.

### Reserve of Nurses.

From a Civilian's Point of View.

The scheme for forming a "Reserve of Nurses for Army Service" is set forth as one in which Princess Christian is personally interested and over which it is proposed that she should be presently nominated the "Superintendent-General." Under the auspices of so popular a leader the movement must obtain favour and even a certain amount of popularity. Probably modifications of the present "regulations" will, however, be suggested to Her Royal Highness, as it is obvious that the primary project of forming a reserve from amongst nurses actually engaged in hospital work will neither coincide with strict justice to the sick poor nor conduce to the successful administration of civil hospitals.

Much needless inconvenience must result from the maintenance on a nursing staff of a number of trained nurses pledged to respond at any moment to a call to another sphere of duty. Even the best of ward sisters would deteriorate if she lived in constant anticipation of a sudden summons to turn her back on her daily work. The single-mindedness which is an essential constituent in a good nurse must disappear before two conflicting claims. It is easy to see why committees who value the reputation of their nurse-training school cannot countenance a request which forms a feature in these regulations: "The respective hospital authorities shall be asked to agree that no nurse shall forfeit her position or prospect of promotion through absence on army service." Surely a reserve drawn from women experienced in military nursing and in foreign service would be more suited for dealing with emergencies than the ward sister who, admirable in her own department, has no knowledge of the difference between her regular work and that of nursing soldiers. Yet there will never be any lack of volunteers eager to smell powder and succour the wounded, and these should receive all the supplementary training which R.B.N.A. or any other asssociation offers them. Reserve nurses, as doubtless Her Royal Highness will shortly learn through those best qualified to advise her, could best be recruited from amongst the unattached community of trained nurses and never from the ranks of ward sisters and staff nurses who are already fully engaged in equally noble if less glorified ministrations to sick and destitute non-combatants.

Doubtless the scheme of Lieut.-Colonel Evatt countenanced by the Princess Christian may be quite easily revised. With due consideration to the claims of the hospitals on the permanent services of their staffs, recruits will be easily raised from the ranks of those who are engaged in nursing outside the hospital wards, although their enlistment will necessarily give the promoters more trouble than the simple expedient of withdrawing tried and trusted workers already associated with regular hospital employment.

### A Case for Assistance.

Miss Bell and Miss Farrow write from the Liverpool Homes for Aged Mariners, acknowledging gratefully the insertion in The Hospital of their appeal on behalf of a nurse, and sending the following list of additional subscriptions: H. L. W. (second subscription), 15s.; "A Nursing Sister," 7s. 6d.; Nurse Griffiths, 3s.; N. L., 1s.; N. H., 1s.; N. K., 1s.; making altogether up to date £3 7s. 6d.

### Wants and Workers.

*H. G.* will be glad to hear from anyone willing to share expenses of taking in *The Lancet*.

Would anyone please tell a District Nurse of any home or hospital for cases of phthisis in the South of England, where a boy five years old could be taken in? Case urgent. Disease not far advanced. Small payment would be made.

### Home and Hospital for Jewish Incurables, Victoria Park Road.

There was an air of cheerful expectancy about the region of Hackney and Victoria Park on the afternoon of Friday, July 20th, for the Duke of York was at Bethnal Green, where he formally opened the "Meath Garden," hitherto known as the Victoria Park Cemetery. From thence His Royal Highness proceeded to visit the Jewish Home for Incurables in Victoria Park Road. A typical and orderly East-end crowd had assembled around the little hospital, where a most cordial welcome was extended to all guests by those deputed to receive the Duke on the threshhold. These included the President, Mr. Raphael, the Rev. Dr. Adler, Chief Rabbi, and Mr. Isaac Davis. As soon as His Royal Highness appeared he was conducted to the Board Room, where a few words of welcome were spoken by Mr. Raphael, who said that the honour of a visit from His Royal Highness was appreciated not only by the immediate friends of the hospital but by the whole Jewish community. He ventured on their behalf, to congratulate the Duke upon the birth of a Prince. The Duke of York, in reply, said that he was pleased to visit the Home, and he thanked them all sincerely for their congratulations on the birth of his little son. The Duke was then conducted over the Home, speaking cheerily to the patients, and regretting that he could not avail himself of the suggestion that he should remain to hear prayers read in the little synagogue in the garden. He finally took his departure amidst hearty cheers from the bystanders.

This little hospital has been established about four years, and is the only institution of its kind in London. It appears to be doing good work amongst the large Jewish population of the East End. Much credit is due to its energetic founders, amongst whom the most prominent are Mr. Raphael, Mr. Barnett, the hon. secretary, Mr. Gollancz, Mr. Drukker, and Mr. Spiers, who have started and maintained the home on a self-supporting system. The subscriptions are in most cases penny-a-week payments from the poorer members of the community. The committee are anxious to raise sufficient funds for the erection of a new and more appropriate building, the present home being merely two old and not very convenient adjacent houses. Although quite the best has been made of the existing accommodation greater conveniences are much needed. Considering the wealth of the Hebrew community surely plenty of support ought to be forthcoming for this little institution.

Every effort is made to make the hospital truly a "home," and the wards are as bright and cheerful as circumstances will permit. On the first floor are the two principal wards, one for men and one for women, with a door opening between them. Upstairs another ward is used for serious cases. A small garden at the back is pleasant for patients able to sit out in fine weather, and the number of wheel chairs, both in use and stored in what Dr. Tunstall, the kindly medical officer, calls the "coach-house" in the basement, show how many attempts are made to brighten the days of the incurable inmates. "Incurable" seems, after all, hardly the right word to use, for no less than three patients now in the hospital are said to have been given up as hopeless cases at other institutions, and are far on the road to entire recovery here. The staff, with the exception of the cook, who must necessarily be a Hebrew, consists entirely of Christians, and includes a matron, Sister Anstey, whose care for those under her charge and devotion to her work meet with appreciation on all sides, one fully-trained sister, and five probationers. Dr. Tunstall, whose name is known in connection with the St. John Ambulance Association, takes what one of the committee called a "fatherly" interest in patients and staff, and gives lectures to the nurses. A room on the ground floor is the nurses' sitting and mess room. It is provided with a piano, which is in much request for concerts for the patients; folding-doors opening from the nurses' sitting-room into the day-room of the inmates enable the double rooms to be utilized for concerts. The committee are very desirous of admitting children, but with the limited means at their disposal this is impossible.

In spite of new wall papers, and flowers and plants requisitioned for the occasion of the Royal visit, the present houses are most inadequate and unsuitable for the purposes to which they are put.

The patients being in most cases very helpless, have to be conveyed in wheel chairs up and down the lift to the garden and day-room. All this heavy work falls upon the nurses—as no man is employed upon the premises. When a helpless patient arrives at the home, young women have to receive and transfer him to the bed allotted to his use.

The project of making the institution a true "home" to these foreign Jews is carried out so literally that the patients are permitted to pervade every part of the house. Passing through the nurses' sitting-room to their own, and intruding on the Matron with equal confidence.

The staff is fully adequate to the number of patients, and yet their work seems never done. Observance of certain services after sunset at this time of the year naturally makes the hour for retiring to bed a very late one. Most of the patients have to be undressed and attended to exactly like children, and many of the attentions required by the men could be discharged with far more propriety by male attendants than by the young women who receive their first training in sick nursing amongst these foreign Hebrews of both sexes. The patients are obviously allowed to have their own way in everything, and amongst so many chronic patients the early days of an untrained probationer can hardly be easy, for "the experienced inmate" is always one of the greatest trials which the novice has to face. It must need all the kindness which the patrons of the institution lavishly bestow on the staff to reconcile the latter to their daily round of duty.

The patients are very strict Jews and there are endless points to be remembered and observed by the woman who wishes to become a popular nurse amongst members of the community. A tone of great kindness pervades the little Home in Victoria Park Road. Everyone bears testimony to the liberal provision of all surgical appliances ordered by the doctor, and of every little luxury desired by the patients.

The courteous Matron and nurses receive every consideration from the appreciative committee, and this doubtless lightens what must be, under all circumstances, monotonous and exhausting work. In constructing a new and more commodious home, we should venture to suggest to its patrons the advisability of their considering the propriety of the male wards being nursed by men. This plan would possibly be a little trouble to organise at first, but it works well in other establishments for chronic cases, and, without entering into all details, we cannot refrain from condemning the practice of leaving such duties in the hands of young women. The massage, from which so much benefit is said to be derived by the patients, and the transport of the latter from floor to floor would also be efficiently discharged by the male nurse, whose training and services are too little accounted of in England.

The probationers are lodged in rooms at the top of the house, each containing two beds, and their only sitting-room is in the basement where they take their meals. There is no possibility of a worker feeling "off duty" in such an apartment, as it communicates directly with the kitchen, the patients' day-room, and the garden where the inmates are always stationed in good weather.

For the sake of the nurses who appear so cheerful and patient amongst a class of patients who must tax their resources to the utmost, we sincerely hope that the wealthy and liberal Hebrew community will shortly see their way to building a home to meet all reasonable requirements.

☞ For Everybody's Opinion, Nursing in Germany, Book World for Women and Nurses, &c., see page clxxix. et seq.

### Everybody's Opinion.

[Correspondence on all subjects is invited, but we cannot in any way be responsible for the opinions expressed by our correspondents. No communications can be entertained if the name and address of the correspondent is not given, or unless one side of the paper only be written on.]

### THE MATRONS' COUNCIL.

### An Inquiry.

"MATRON" writes:—I enclose a letter I have received from Miss Stewart. It runs as follows: "St. Bartholomew's Hospital, London, E.C. Madam,—It was decided at the preliminary meeting of the Matrons' Council, held here on the 13th of this month, that you should be asked to become a member of the Executive Committee. I hope you will be able to do this. Our first meeting will be held here on Thursday, 20th July, at 4.30 p.m., which hour, you will, I hope, find convenient.—Yours faithfully, (Signed) ISLA STEWART, Matron and Superintendent of Nursing (Reg. Nurse)." Do you think the Matrons' Council will be a party affair? I apologise for troubling you, but, knowing you have this nursing question at heart, I venture to write to you. As far as I can judge, only registered nurse matrons were represented. Do you think it would be wise to join them, or to become a member of the Council? I shall be obliged for any help you can give me in the matter.

*** Our correspondent, who is one of the most successful of matrons, may have unconsciously answered her own question. She says that "only registered nurse matrons were represented," and, if this was so, then the meeting clearly consisted of members of one party only.

### By a Matron who was Present.

I have found that the disadvantage of living in the country is that one does not hear or know of anything that goes on in the London nursing world, hence my reason for attending the Matrons' Council. I found from the first that there was a decided party feeling at the meeting; Mrs. Fenwick or Miss Stewart either promptly sat upon or ruled out of order anyone who raised obstacles; in fact, a Miss Clark (I believe that was her name), who objected to the name of "Matrons' Council" if Sisters, &c., were admitted, was ruled out of order six times at least. As far as the meeting went during the time I was there, there was decided opposition, but in courtesy to Miss Stewart, who was in the chair, and the meeting being held in her own hospital (also, she had asked for consideration as she had the matter at heart), the last propositions that were put to the meeting the "opposition party" would not vote for at all. I then left, saying to the lady sitting by me evidently it was a party scheme and I should not wait any longer; it was waste of time; also, no accurate feeling could have been arrived at, as during the whole of the first rules, the Sisters, Charge Nurses, &c. (a great many of whom were present from the Bart.'s staff), voted for and with Miss Stewart. I have no intention of joining the Matrons' Council. I saw by THE HOSPITAL on Friday that you had no representative there; that decided me to write you.

### Another View.

I attended the meeting at St. Bartholomew's on purpose to gather information, and not with the intention of joining the Matrons' Council at present. I send you the papers I have as you may like to see them. I wanted to see who were the matrons interested in the proceedings. They were a little late beginning, and I left early. During the discussion it seemed that no effective opposition would be allowed, and some had evidently studied each paragraph, especially No. 2, which refers to a subject not sufficiently valued by women. Any object which would or could be advanced to bring matrons together would have my sympathy, and I should prefer the association embracing matrons only. Still I feel that we should do something; "old things are passing away." It was a great and possibly a successful meeting, but I had my doubts about future success and continuance of the movement, if some associated together seven or eight years ago had too much influence in this new venture. It was a surprise not to see at the meeting such well-known matrons as Miss Wood and Miss Ingall.

A HOSPITAL MATRON OF SOME YEARS' STANDING.

### A Lesson from the United States.

A correspondent writes: Americans are duly sensible of the many advantages which the old country possesses over the new. It appears to them, however, that in nursing matters the United States shows most wisdom and common sense. Instead of a few matrons rising up in America, and starting a nurses' association on their own account, as some of your disaffected ones have done, we had a meeting two years ago, when the matrons of all the big hospitals including all shades of opinion were represented, at which it was determined to first organise the schools by securing uniformity of training; then to gradually create alumni associations in connection with each school and large hospital; and then perhaps—but not till then—we shall be in a position to establish an American Nursing Association, which will really deserve the name. Miss Isla Stewart seems to be making a grand mistake in regard to her proposed Matrons' Council. In the United States the matrons have started a superintendents' association, but have confined its membership to the women who are at the head of the nursing in our largest hospitals, to the exclusion of those who are in charge of the special hospitals and smaller institutions. This step was taken in order to secure a proper basis on which to build an American Superintendents' Association, which should include only those superintendents who were entitled by their training and position to be admitted to membership. Judging from the list of those reported to have been present at St. Bartholomew's Hospital on the 19th instant, it appears that, with a few exceptions, the superintendents of the great British hospitals were conspicuous by their absence. If the Matrons' Council or Superintendents' Association is ever to be successfully established in Great Britain, Miss Isla Stewart must follow the American precedent and confine the membership at the outset to the matrons and superintendents of the larger general hospitals which have training schools attached. These superintendents, from their knowledge and experience, may be able—no one else can—to elaborate a scheme for such an association which would commend itself to general acceptance. Experience in the United States proves that any attempt to establish a Matrons' Council without the co-operation of the superintendents of the great hospitals throughout the country, to whom the movement should be initially confined, must prove unsuccessful, because it will lack the essential elements, without which success is impossible. Is it too late for Miss Stewart to reconsider her position, and to set about securing the co-operation of the leaders of nursing in England? If she has attempted this and failed, I hope she will let the matter rest for the present, and so decline to put herself at the head of yet another "schism" which cannot fail to prove harmful rather than helpful to the best interests of nursing in the old country.

### HEALTH LECTURING IN RURAL DISTRICTS.

"Examiner" writes: In the recent article on the above subject, "Lecturer" seems inclined to be somewhat sarcastic in referring to the St. John Ambulance certificates, the result, probably, of being unacquainted with the amount of theorectical and practical knowledge required to obtain them. Having had an opportunity of comparing the St. John's course with one given by a trained nurse, I must say the result was very much in favour of the former, for these reasons: For the St. John's nursing certificate a student must first obtain a "first aid certificate," thus gaining a practical knowledge of methods of affording relief in many cases, and after attending a course of lectures on nursing, may present herself for examination, when in addition to having a written paper to answer, she will have to show a thorough practical knowledge of the following: Reading the clinical and ordinary thermometer, making temporary bed-rests, splints, and cradles; application of tourniquets to main arteries, treating fractures, moving patients, changing sheets, &c. In the trained nurse's courses, lectures were given to about a hundred students; practical work was shown from the platform by the help of one or two students, and at the end of the series a written examination is held, no practical work being required. When a "hospital-trained" nurse cannot be obtained, a student holding a St. John's certificate would be far more useful than one who had gained their experience from the "nurse's course" as at present carred out.

[I doubt much if "Examiner" would like to be nursed by a student holding St. John Ambulance certificates, but having no other experience of nursing. I by no means wished to disparage these certificates, for, no doubt, those holding them have gone through much useful work. Yet "first aid" is not nursing, and as the nursing course is given for the St. John's society by a doctor, the student learns no nursing from a nurse's point of view. Doctors give orders, but nurses carry them out, and in my opinion there is ample employment for both. Few doctors would care to have to carry out their own orders, however competent to do so. When an experienced trained nurse lectures she is speaking of what she herself has actually done in a sick room (not only on a platform), and if her facts are less numerous they are certainly more practical than those of a medical man who lectures on nursing. On more than one occasion I have had the opportunity of comparing the knowledge gained from a St. John's course and that gained from a course of lectures given by a trained nurse and lecturer, and have found the latter is the more useful. We hear much in these days about nurses not treading on doctor's ground, and I have no wish to encourage an intrusion which ought never to be perpetrated by any fully-trained women.—A. E. I., Lecturer.]

### BATHS.

A. F. Jones writes: Is it desirable to take a cold bath when hot after exercise, or should it be tepid? I should be glad to have the opinion of some of your correspondents on this subject, which seems to me one of general interest.

---

Mrs. Bedingfeld writes: Seeing a query (97) in The Hospital for July 14th regarding a lunatic asylum in Norfolk, I beg to inform "Inquirer" that Heigham Hall, mentioned in answer, is a private asylum, only suitable for patients able to pay. My father, the late W. H. Ranking, M.D., F.R.C.P., of Norwich, was for many years prior to 1864 part proprietor of Heigham Hall. The county asylum at Thorpe, a suburb of Norwich.

### LONDON OBSTETRICAL SOCIETY.

The July examination of this society was successfully passed by Sister Winnifred, Sister Hilda, and Sister Emily, of the Community of St. Lawrence, Belper.

### Nursing in Germany.

The General Assembly of the "Vaterländischen Frauen Verenis" was held this year at Berlin, and was honoured by the presence of the Empress Victoria, whose interest in nursing is well-known. The report ot the society shows that it has accomplished a large amount of work during the past year, and has increased the number of branches from 782 to 804. It is very prosperous, its annual subscriptions and income amounting to no less than six and a-half millions of marks (£325,000), which is chiefly devoted to sick nursing. Eight branches have erected and entirely support large hospitals which have homes for the nursing sisters. At the present time there are 1,048 trained nurses, and of this number 458 are deaconesses, 23 are sisters, 384 sisters of the Red Cross, and 183 belong to no particular order. In Berlin a new branch has recently been formed to erect a hospital and train nurses. According to a recent statement of Dr. Menger there are sufficient temporary hospitals to afford accommodation for 200,000 sick and wounded. These are ready for use at any time, and would be available in case of either an epidemic or a war; they are provided by the Voluntary Sick Nursing Association. During the Franco-Prussian War the Red Cross Association provided accommodation for 75,000 wounded. A new hospital is being built in the northern quarter of Berlin. Already a number of houses have been pulled down to make room for it. This will make the fourth general hospital in the capital.

### Appointments.

Poplar and Stepney Sick Asylum.—Miss Sarah Ann Hannaford has been made Matron of the Poplar and Stepney Sick Asylum. She was trained at the Western Infirmary, Glasgow, and Whithington, near Manchester. Miss Hannaford has also held the appointment of Matron at Chorlton Union Hospital, and we wish her every success in her new work.

Southampton Free Ear and Eye Hospital.—Miss Fanny Warren, who was trained at the London Hospital, has been appointed Matron of this hospital. During five years Miss Warren filled the posts of Nurse and Matron at the Kendal Memorial Hospital. She was then Night Superintendent at the London Fever Hospital, and subsequently Matron of Hitchin Infirmary for three years. We wish her every success in her new work.

### Minor Appointments.

Stockton-on-Tees Fever Hospital.—Miss Margaret Martin has been appointed Matron of this hospital. She was trained at the City Hospital, Edinburgh, and was afterwards Fever Staff Nurse at the Royal Infirmary, Perth. She takes many good wishes with her to Stockton.

### Notes and Queries.

**Queries.**

(102) *Monthly Nursing.*—I should be glad to know the names of lying-in hospitals in or near London, where the training is short, but good.—*Sister Marion.*

(103) *Maternity Hospitals.*—Can you tell me of any maternity hospital where probationers get board and salary or board alone for their services?—*Nurse M.*

(104) *Nice.*—Will you kindly send me the English address of the Superintendent of the Hollond Institute?—*A. B. B.*

(105) *Cannes.*—Please give me addresses of nursing homes.—*Eleanor.*

**Answers.**

(102) *Monthly Nursing* (*Sister Marion*).—Under the head of "Nursing," you will find the information you require in "Burdett's Hospital Annual," published by Scientific Press, 428, Strand. The Hon. Secretary of the Midwives and Trained Nurses' Club, 12, Buckingham Street, Strand, is very kind in giving advice, but if you write to her you should send a stamped and addressed envelope for reply.

(103) *Maternity Hospital* (*Nurse M.*).—See previous answer.

(104) *Nice* (*A. B. B.*)—Miss Woodcock, All Saints' Vicarage, Axminster.

(105) *Cannes* (*Eleanor*).—See "Burdett's Annual" and letters from correspondents.

### The Book World for Women and Nurses.

[We invite Correspondence, Criticism, Enquiries, and Notes on Books likely to interest Women and Nurses. Address, Editor, The Hospital (Nurses' Book World), 428, Strand, W.C.]

Manual of Hygiene. By J. White Wallis. (London: Kegan Paul, Trench, and Co. 1894.)

This manual has avowedly been written to fill a vacant spot in the educational literature of the century. No effort, it would seem has been made in England to arrange the subject of public health and its relation to other sciences in a manner suitable for the study and education of children before they leave school; at least, so says the preface. We fear, however, that so far as a reliable school manual of hygiene is one the wants of the age, that want still remains unsupplied, for we can hardly conceive of the book before us being more inappropriately used than by its being placed in the hands of children as a lesson book. Such a book should not consist of a series of more or less contradictory aphorisms, should be a self-explanatory guide rather than a storehouse of conundrums for the puzzlement of teachers, and above all things should be accurate. Hard, indeed, would be the lot of the teacher who, after describing the origin of animal heat, had to explain the following sentence: "Cold-blooded animals are almost entirely dependent on the sun for the heat which is the source of their vital force." Then again we find the following curious assertions coming in rapid succession! "In cold climates, the air being denser, exerts more pressure on the blood vessels of the lungs, and thus more oxygen is absorbed and the heat of the body is maintained." Does the author believe that the pressure of the atmosphere is greater in cold climates, and that this accounts for the greater heat production within the body in such regions? "The loss of heat occasioned by radiation depends on the temperature of the surrounding atmosphere." Surely this is incorrect. Combustion is a great stumbling-block to the author. Among other odd remarks we find the following: "Artificial light should fulfil two important conditions—it should give a suitable light, and should burn the products of its own combustion." What does the author mean? Does he suggest that the water or carbonic acid produced by the combustion of a candle has then to be burned? Having defined gas as a "bicarburetted hydrogen," how is the teacher to explain the fact that "as it burns it gives off a poisonous gas which soon vitiates the air," while on the opposite page, lighting by oil, also consisting of carbon and hydrogen, is said to be "convenient and healthy"? Of course, one sees what is meant; but why leave it all to be explained? Again, why speak of "oxyhydrogen gas" among the illuminants, as if such a substance existed, instead of using the term "oxyhydrogen light," which we presume is what is meant. We all agree that "mineral oil must never be used to quicken a fire," but the assertion would be more interesting and more instructive if accompanied by an explanation. The following definitions in regard to electricity are neat but not very explanatory, and must surely involve the teacher in a veritable quagmire of deep science if he is to make their meaning clear. "Positive electricity is the electrical condition of the atmosphere in clear fine weather." "Negative electricity is the electrical condition of the atmosphere in damp and stormy weather." Under the heading "Precautions to be Taken During a Storm," we are told that "buildings of any considerable height should be supplied with lightning conductors." Of course, nobody dreams that the author means this to be done during a storm; nevertheless it is sheer carelessness to place such directions under such a heading. That the author's knowledge of dairy work is not profound is shown by the following curious passage: "When milk coagulates slowly, cream rises to the top, and this, when churned, forms butter. The serum which remains behind (water, lactine, lactic acid, and salts) is skim milk." The effect of winds upon the human frame seems to be of a striking and disastrous nature. "The warm, humid winds are depressing; in such an atmosphere the functions of the skin and lungs are hindered or suppressed, and too great an activity is forced on the loins and mucous membrane." We should have thought that if the functions of the lungs were suppressed there would be but little chance of activity either in the "loins or mucous membrane." "If the wind is cold and humid, then less oxygen is absorbed by the system, and there is a slackening of combustion; this entirely disorganises the lymphatic system or tissue growth, the nerves enlarge, the flesh fattens enormously, &c." This is all very alarming, and if judiciously placed before a parent by a child endowed with intelligence and ready wit, will be conducive to many holidays on damp raw mornings in November. More amusing, perhaps, is the account of temperaments and constitutions.

The lymphatic temperament is evidently a fearsome thing, "characterised by predominance of vitality and action in all the tissues penetrated by white liquids, mucous serum, &c." These "elegant extracts" are but snippings. The book is full of good things, likely to be productive of much amusement to the judicious reader, but surely somewhat bewildering to the child at school into whose hands it is put.

Our Manifold Nature. By Sarah Grand. (London: Heinemann. 1 vol.)

The preface tells us that our manifold nature can be traced in these six short stories collected from Madame Sarah Grand's magazine contributions. The reader, however, will find the title somewhat incongruous with what he is led to expect. This is especially the case in the first and longest story of the collection, "The Yellow Leaf." This tale would better serve as an example setting forth the uniformity of feminine nature, in its struggle towards the great goal of a successful marriage, the variation being in the means and not in the end. "The Yellow Leaf" traces the careers of two young women, Evangeline and Adalesa. Evangeline had been brought up in what Madame Grand describes as the "feather-bed" system—and taught to be attractive before anything else. The other, Adalesa, who represents the New Womanhood, might with equal justice have been compared to a flock mattress—and a lumpy one. She is the exact reverse of Evangeline in all particulars. Whereas Evangeline's style is to be pretty and gentle in manner, however despicable in character, Adalesa prefers to adopt a masculine, severely outspoken attitude, and thinks it necessary to carry a bull pup under her arm. They both fall in love with the same man, who forsakes Adalesa for the gentle charms of Evangeline. Adalesa thereupon takes to dressing well, develops into a fine young woman, and marries a duke. To be consistent she should have remained scraggy, become a member of the School Board, and asked nothing better from life than the emancipation of her sex. The other stories are rather dull, with the exception of "Eugenia," which tells of a girl whose choice lies between a hopelessly bad young man, and a hopelessly good young man. She chooses the latter, being a New Woman. Eugenia herself is well drawn, and quite a possible person, but the two men could only exist in the work of a lady novelist. The story of "Ah Man," shows us that sketches of foreign eccentricity have succeeded rather in the telling than in the matter of them, and Messrs. Rudyard Kipling and Bret Harte have dealt with these characters in a way which admits of no less skilled treatment. Apart from the question of literary merit, "Ah Man" has appeared too late in the day to be anything but a failure.

## Figures and Tables

**Figure f1:**
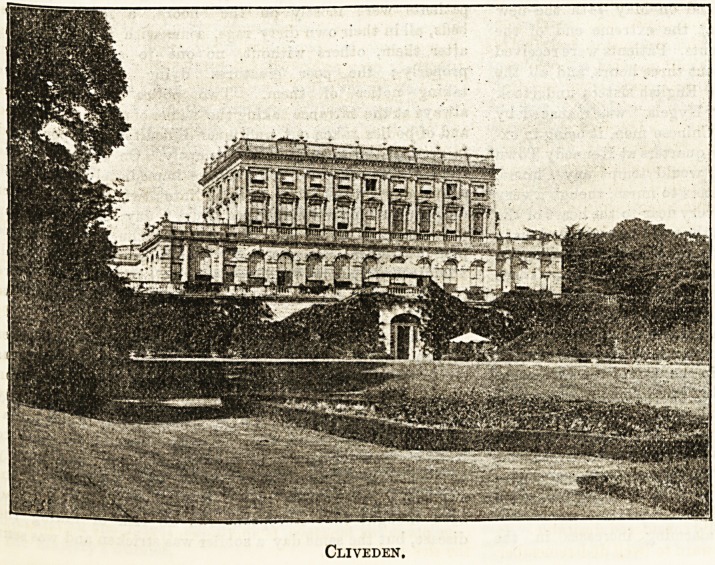


**Figure f2:**